# Cultural poetics of illness and healing

**DOI:** 10.1177/13634615231205544

**Published:** 2023-11-07

**Authors:** Laurence J. Kirmayer

**Affiliations:** Division of Social & Transcultural Psychiatry, Department of Psychiatry, McGill University, Montreal, Quebec, Canada

**Keywords:** embodiment, enactment, healing, illness experience, metaphor, ontology, poetics, psychotherapy

## Abstract

This issue of *Transcultural Psychiatry* presents selected papers from the McGill Advanced Study Institute on “Cultural Poetics of Illness and Healing.” The meeting addressed the cognitive science of language, metaphor, and *poiesis* from embodied and enactivist perspectives; how cultural affordances, background knowledge, discourse, and practices enable and constrain poiesis; the cognitive and social poetics of symptom and illness experience; and the politics and practice of poetics in healing ritual, psychotherapy, and recovery. This introductory essay outlines an approach to illness experience and its transformation in healing practices that emphasizes embodied processes of metaphor as well as the social processes of self-construal and positioning through material and discursive engagements with the cultural affordances that constitute our local worlds. The approach has implications for theory building, training, and clinical practice in psychiatry.

## Introduction

Language is the most distinctive feature of the human mind, with which we fashion our lifeworlds through acts of *poiesis*, giving voice to suffering, making sense of our predicaments, and finding ways to heal. Each language of suffering and affliction is deeply rooted in a cultural history, geography, and community. Every expression of distress and effort at transformation draws from these shared languages even as individuals improvise new hybrid forms. Understanding the creative functions of language must be central to a psychiatry for the whole person. This issue of *Transcultural Psychiatry* brings together papers from the 2019 McGill Advanced Study Institute in Cultural Psychiatry on “Cultural Poetics of Illness and Healing: Embodiment, Enactment and the Politics of Experience.” An interdisciplinary group of scholars engaged in studying how language shapes experience through embodied metaphor, narrative enactment, and situated performance presented work exploring the ways that cultural resources suffuse our linguistic capacity and modes of self-construal in the experience and expression of symptoms and illness and in the processes of identity transformation and healing. Papers presented at the workshop addressed the cognitive science of language, metaphor, and poiesis from embodied and enactivist perspectives; how cultural affordances, background knowledge, discourse and practices enable and constrain poiesis; the cognitive and social poetics of symptom and illness experience; and the politics and practice of poetics in healing ritual, psychotherapy, and recovery.^1^

This introductory essay will outline an approach to poiesis through the study of metaphor in medicine, psychiatry, psychology, and other healing systems. Our own self-understanding through metaphors and narrative constructions plays a key role both in internal regulation and in engagement with our social environments, which are largely constituted by ongoing interactions with others. The language we have available to articulate and express our experience changes the very nature of that experience. This is the case even for seemingly obdurate experiences of pain and suffering, no less than for the stories we borrow or invent to carry on our lives and project ourselves into new and better circumstances. The implication is that an adequate picture of the emergence of illness experience and its transformation through healing practices must lay bare the embodied processes of imagination as well as the social processes of self-construal and positioning through pragmatic, material, and discursive engagements with the cultural affordances that constitute our local worlds and niches. While much of this process of meaning-making is organized by and communicated through narratives, metaphors play a central role in efforts to make sense of symptoms and suffering for both patients and healers (Kirmayer, 1992, 1994, 2000).

## The logic of metaphor

**Figure 1.**
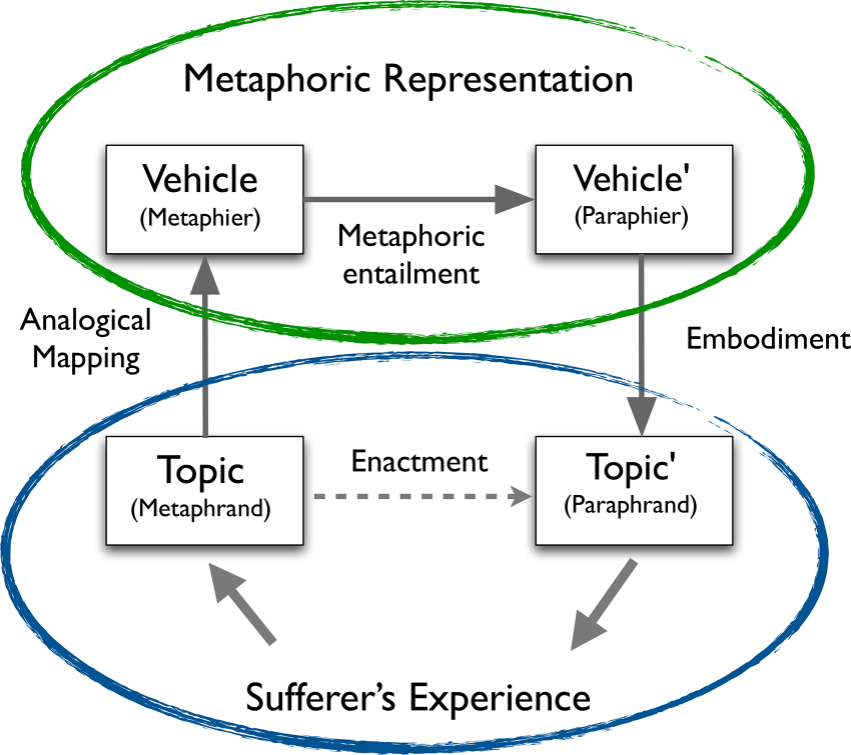
The structure of metaphor in illness experience and healing.

*Note.* Metaphor involves thinking about one thing (a topic or target) in terms of another (vehicle or source). Finding the relevant analogies or facets of source and target involves interaction between the two in a context-driven process of search or imaginative construction. Metaphors can build a scaffolding that links developmentally earlier and conceptually simpler bodily experiences to more complex situations, scenes, or scenarios. The terms *metaphier/paraphier* and *metaphrand/paraphrand* are borrowed from Jaynes (1980). The overall structure has been termed a *transformagram* (Hinton & Kirmayer, 2017). In addition to the cognitive effects of analogical mapping, the key to the transformative effects of metaphor lies in the processes that link metaphoric entailment with embodiment and enactment. Adapted from: Kirrmayer, 2011, p. 117.

Metaphors are mappings between one object or domain (the *source* or *vehicle*) and another (the *target* or *topic*). This mapping confers new meaning on the target and, as the term “vehicle” suggests, the cognitive process of metaphor allows us to extend the mapping to derive additional associations or implications. Although the concept mapping that underlies metaphor is asymmetrical, applying elements of the source to the target rather than vice versa (e.g., surgeons are butchers is not the same as butchers are surgeons),^2^ metaphor comprehension and production involves an interaction between source and target that may alter both, creating new meaning and salience (Sternberg & Nigro, 1983). Metaphors can provide a basis for further metaphoric elaboration, scaffolding more complex or abstract cognition (see Figure 1).

At the base of the scaffolding, metaphors provide semantic pointers to grounded meanings.^3^ This implies a direct mapping from source onto target. But metaphors typically present polyvalent dimensions of meaning that point in multiple (sometimes discordant) directions or that may lie dormant, waiting to be activated in surprising ways. Many metaphors are part of conventional idioms or expressions that have become the literal name or description of an object or event. Conventional metaphors provide reassurances that we are participating with others in the same language game, the same community or form of life, with its familiar etiquette, idioms, rules, norms, and implicit ontology. The use of a novel metaphor (or of a conventional metaphor in a novel way that reactivates the meanings inherent in its etymology or experiential grounding) is an invitation or provocation to think anew about some aspect or domain of experience. This is a basic way we generate new ideas, actions, and perceptions that extend the boundaries of cognition and culture.

The metaphors we use to make sense of experience can be systematized as schemas or models based on analogical mappings. To the extent a metaphor is systematized, it can become a model of experience but, unlike a formal analogy or explicit mapping, the metaphor need not conform to its initial target domain, and this may lead to divergent meanings. While analogies may provide the basic structure or mapping that prompts metaphor, metaphors have excess or supplementary meaning that is not captured by a formal analogy. In effect, metaphors prompt a search for additional meaning—the more novel or strange, the wider or deeper the search. This search may involve picking out one or other aspect of the source to attach its quality (or entailment) to the target, or it may involve constructing images, scenarios, or possible worlds in which the metaphor makes sense.

A metaphor can point to an explicit analogy or model that aims to capture part of the mechanism—the actual process that subserves a particular phenomenon. It may re-orient our perspective on a situation or event by drawing attention to neglected facets or imbuing it with new meaning and connotations. But metaphors can also be mechanisms themselves: machines for the generation of meaning in ways that are highly consequential. Viewing a person in terms of a sensory metaphor (e.g., “she is a warm person”) modifies our way of relating. Evocative metaphors provided by psychiatry and psychology like “broken brains,” “chemical imbalances,” “trauma,” and “resilience” can reshape our sense of agency and subjectivity. In research, thinking about the brain as a computer (von Neumann, 1958) has motivated a vast body of work in computational neuroscience and psychiatry, despite the limitations of the metaphor (Brette, 2022); at the same time, thinking about one's own brain as a computer leads to modes of self-reflection that can change our way of being in the world (Martin 2010; Rose & Abi-Rached, 2013; Vidal & Ortega, 2020).

To the extent that metaphors offer new ways of looking at familiar objects or events, they may be viewed as cognitive tools or gadgets (Heyes, 2019). New metaphors can be readily fashioned, yielding new tools. In this way, metaphor provides a highly flexible, granular, modularity of mind. In a sense, metaphors function as temporary cognitive modules assembled to do specific types of information processing, regulation or transformation of action and perception. Each metaphor becomes a jury-rigged module that serves the generative/constructive nature of cognition. But metaphors have lives of their own, both cognitively and socially. As a cognitive tool, every metaphor has its own affordances and limitations that leave marks or traces on the concepts and experiences it is used to transform. Socially, every metaphor provides a perspective or way of seeing the world, framing situations, identifying goals, and organizing action for individuals and groups (Schön, 1979). Crucial to this autonomy and constructive power of metaphors is the extent to which they can be stabilized and ramified. This stabilization can occur through a metaphor's connection to embodied experience or discourse and by its embedding in a web of social practices and institutions. This points to the need to understand metaphor in terms of the dynamics of social-ecological contexts (Gibbs, 2019).

## Embodiment and enactment

**Figure 2.**
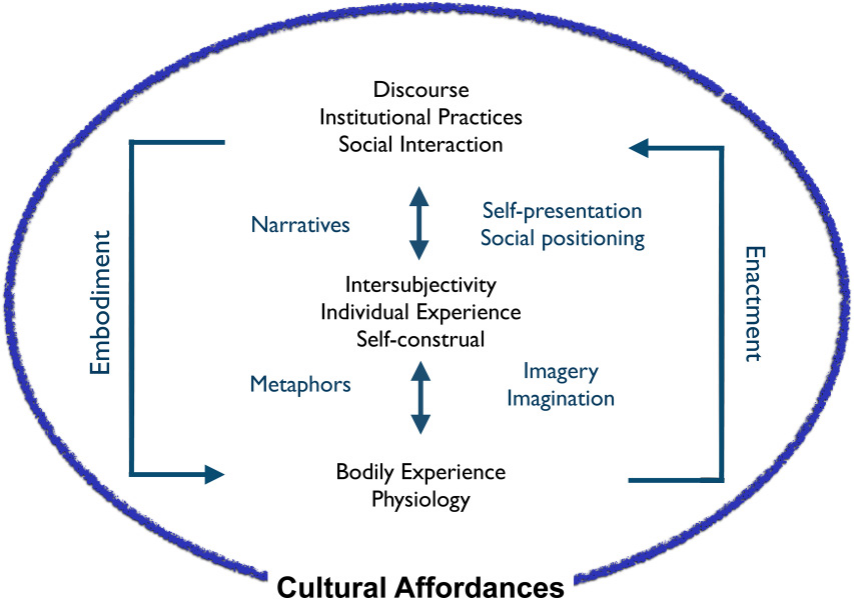
Embodied and enactive processes of meaning making.

*Note*. The figure outlines the cyclical processes of embodiment and enactment that mediate experience. There is two-way traffic between bodily processes and individual experience mediated by cognitive processes of metaphor, imagery, and imagination. Similarly, there is traffic between experience and social discourse and institutional practices mediated by interpersonal communication through narratives, self-presentation, and positioning. All of this occurs in a field of cultural affordances provided by local niches and larger social contexts. Based in part on Figure 20.1 in Kirmayer & Ramstead, 2017, p. 406.

Explanations of illness experience typically focus either on physiological, psychological, or social processes, but there is a need to bridge these coexisting dimensions. Metaphoric embodiment breaks down this distinction by showing the links between bodily and discursive processes. Figure 2 outlines the dynamic relationship between individuals’ subjective and intersubjective meaning-making and its dual grounding in bodily experience and in social-cultural discourse and practices.

This grounding can be understood through current work in 4E cognitive science, which views cognition and experience as essentially *embodied*, *embedded*, *enacted*, and *extended* (Newen, De Bruin, & Gallagher, 2018). The notion of embodiment draws from the work of the phenomenological philosopher Merleau-Ponty (1962; Hass, 2008), but now includes experimental work on grounded cognition, metaphor, and social physiological interactions through which the body shapes cognition and provides natural metaphors for experience (Barsalou, 2010; Gallagher, 2006; Gibbs, 2005; Johnson, 2013). There is two-way traffic between bodily anatomy and physiology, cognition, and social discourse. *Embedding* refers to the fact that cognition and experience are always situated in material contexts of body and environment and depend on this embedding for their content and dynamics. *Enactment* emphasizes the fact that cognition occurs in and through action—that is, active engagement with the environment, which in the case of humans involves cooperative activity to construct social and cultural systems. Embedding and enactment necessarily entail a view of cognition as *extended* into the environment in profound ways such that the most basic unit of cognition becomes a kind of loop between individuals and their local world or niche. In the case of humans, this niche is constructed and constituted by relationships with others (Veissière et al., 2020).

Our embodied experience and interpersonal interactions depend on material and social structures but also on modes of self-construal and self-presentation through language (Wilson, 2022). The ways we understand ourselves and others are central to our navigation of the social world and response to adversity and illness. This process of self-understanding is mediated by metaphors and larger narratives (Hagberg, 2019; Pritzker, 2007; Ricoeur, 1974; Schechtman, 2018). Metaphors and their narrative elaboration thus serve both cognitive and interpersonal functions. This can be modelled in terms of processes of active inference, in which linguistic processes serve a broader adaptive function of predicting the future or imagining possible situations we can anticipate or avoid through action (Bouizegarene et al., 2020).

Active inference shows why we mainly see the world as we expect it (overlaying our model on the stream of sensory input) and notice only substantial differences and discrepancies (measured against our expectations, goals, and limits of tolerance) as “surprises” that warrant deeper processing (Clark, 2015; Parr et al., 2022). To re-establish our predicted regime, we can either act on the world (moving or changing it in some way) or update our models, expectations, priors, and predictions.^4^ Language and poiesis provide cognitive and communicative resources to co-construct and navigate the social world. A computational theory of metaphor would allow us to include this creative function of poeisis at the center of our understanding of the mechanisms of psychological adaptation and psychopathology (Constant et al., 2022).^5^

Although it is not difficult to give a generic account of the cognitive semantics of metaphor in terms of active inference, these models leave out the density of meanings that reside not simply in an individual's environment but in the long history of stories, norms, and discourse that depend on cultural institutions and practices. In active inference models, these layers of situated meaning are collapsed into expectations or priors. The history lies hidden in a set of variables that must capture broad networks that extend over cultural and geographic space and time. Unpacking the origins and ongoing dynamics of these priors as lived realities requires tracing specific connections between cognition, language, and the social world. Hence, social history is essential to understanding the ways that local metaphors mediate individual and cultural meaning.

## Conceptual metaphor theory and the scaffolding of experience

Metaphors are ubiquitous: studies of natural language find about one metaphor for every 10 to 25 words in everyday talk (Cameron, 2008) and about one metaphor for every 25 words on TV (Bowdle & Gentner, 2005). Situations that are affectively charged or in which people struggle to describe unfamiliar or inchoate experiences tend to have still higher rates of metaphor use. For example, in psychotherapy, patients may produce about six metaphors a minute (Gibbs, 1994). Although metaphor was classically approached as a matter of linguistic, poetic, or rhetorical figures that add color, emotion and rhetorical power to speech, work in cognitive linguistics over the last four decades has provided evidence for the centrality of metaphor in thought (Lakoff & Johnson, 1980; Gibbs, 1994). In this view, in addition to their obvious aesthetic and rhetorical functions, metaphors provide conceptual structures that organize perception, cognition, emotion, and action (Gibbs, 2017; Kövecses, 2020).

In this issue, Raymond Gibbs, Jr. (2023), a leading figure in the experimental study of metaphor and the development of conceptual metaphor theory, presents an overview of current thinking about the role of metaphor in cognition and its implications for our understanding of illness experience and healing. He distinguishes between metaphors as figures of speech and conceptual metaphors which underlie abstract thinking. People understand complex or novel phenomena by using metaphors drawn from bodily experience or other familiar domains. This involves processes of bodily simulation and imagination. These simulation processes reflect the body's anatomy and physiology, but they also have a developmental history and cultural embedding. Hence, they depend on cultural narratives, models, and practices (Kövecses, 2015). We acquire metaphors for experience early in development as we interact with the world in ways that are emotionally charged and situated in relationships and social contexts. These early metaphors are then applied to think about new experiences and extended or *scaffolded* to grasp more complex phenomena and abstract concepts (Varga, 2019). Our metaphors thus have built into them our individual developmental trajectories and aspects of cultural history that are sedimented in the etymology and associations of specific words and images. Each of these sources of metaphoric meaning and connotation may have unique facets that depend on local histories and contexts and shared aspects that reflect biological, developmental, social, or existential universals.^6^

Given this intertwining of personal development and cultural history, close attention to metaphor can help us appreciate subtle differences in the ways that individuals from different cultures (and with different life experiences) conceptualize and narrate their experience.^7^ For example, the metaphor life is a journey builds on basic experiences of moving along a path but is elaborated by a wealth of personal and collective experiences with journeys—large and small. The metaphor healing is a journey is common in discussions of trauma therapy and recovery and reflects the ways that growth and transformation require effort and movement over time, meeting challenges and surmounting obstacles to arrive at a new place (Scott et al., 2017). The healing journey metaphor has salience for Indigenous peoples because of cultural notions of the person as connected to the land, travelling along familiar or new paths to find subsistence and resources, and navigating the transgenerational impacts of the long arc of colonization and postcolonial survivance (Lane et al., 2002; McCormick, 2009; Waddell et al., 2021).

Some metaphors are novel while others are well-worn and familiar. The status of any given metaphor depends on the speaker, listener, and the local social context. Familiar or conventional metaphors may be quickly understood by others, invoking common models, and mobilizing shared associations. When metaphors are completely conventional and seem to have lost their generative function, they may be described as “dead”—not recognized as metaphors and treated simply as a label, conventional name, or literal description like the “lip of a bottle”—yet their metaphoric connotations and entailments can be easily reactivated and, even when not deliberately activated, may influence conceptual thinking, imagery, and affective response (Lakoff, 1987; Gibbs, 2017). Unfamiliar, surprising, or novel metaphors provide new ways of thinking and new possibilities for action and perception—and may challenge conventional views by calling attention to the possibility of radical reframings of experience.

There are many common metaphors found across diverse cultures and languages (Kövecses, 2005). Some of these common metaphors have their roots in “physiognomic perception”—seeing the world as like the body, e.g., angry thunderclouds, smiling sunbeams, placid lakes (Marks, 1996). Such metaphors represent “low level metaphorical associations between concepts based on experiential correlation” (Grady, 1999, p. 24). For example, metaphors linking anger with heat (“hot-headed,” “burning, simmering, or boiling with rage”) occur in very different languages and presumably reflect an embodied experience and metonymic connection: anger is commonly associated with flushing and feelings of heat in the face or head. This metaphoric association is found across disparate cultures, even where emotion lexicons vary substantially (Matsuki, 1995; Yu, 1995) and reflects the physiology of anger (Wilkowski et al., 2009). In contrast, loneliness, loss of social connection, and isolation may be described as cold (IJzerman et al., 2012). But of course, flushing also occurs with embarrassment or sexual arousal or other forms of excitement. And anger, while built around an adaptive response to threat, is elaborated and reconfigured in very different ways in different cultures, so that neither the bodily experience nor the emotion is precisely the same (Barrett, 2017). Thus, anger may have very different connotations within and across cultures depending on this larger embedding in emotion dynamics and social scripts—in some instances, for example, we might characterize an angry person as cold and distant, or scheming and predatory (the association is with cold-blooded predators).

Other facets of bodily experience can also provide natural metaphors. Some smells are associated with disgust, and hence the notion that i smell something rotten is a metaphor for suspicion follows readily from this biological preparedness to detect dangerous decay or infection (Curtis, 2013; Ibarretxe-Antuãno, 1999). More complex metaphors may also be grounded in early experiences and built up through processes of scaffolding (Williams et al., 2009).

## Articulating and elaborating illness experience

Medical semiotics tends to assume that there is (or ought to be) a one-to-one relationship between physiological events and symptom reports. In fact, symptom reporting is mediated by attentional, affective, and interpretive processes as well as interactions with others. This plays out in everyday experience through processes of embodiment and enactment that are mediated by metaphors and larger narrative structures. Illness cognition models consider the ways that we attend to and interpret bodily sensations as well as our own thoughts and feelings as the mediators of symptom experience (Kirmayer, 2008).

Recognition of cultural variations in illness experience has led to efforts to rethink the nature of symptoms as not simply signs of underlying physiology or psychopathology but as cognitive efforts to make sense of experience and communicative acts to convey concerns to others and mobilize help (Kirmayer & Young, 1998). DSM-5 incorporated some of this thinking to understand cultural variations in symptom experience (Lewis-Fernández & Kirmayer, 2019). In place of the longstanding notion of culture-bound syndromes that was based on culture-specific symptoms, DSM-5 introduced the notion of *cultural concepts of distress*, which may include syndromes, but that more commonly involve cultural idioms of distress, explanatory models, and folk categories of illness that are part of local ethnomedical systems. This is an important shift away from interpreting the language of distress as always pointing toward discrete disorders toward a focus on the pragmatics of communication in social and cultural context.

Many cultural idioms reflect metaphors that have become codified or routinized in everyday speech. Gibbs (1993) points out that while idioms may become formulaic and be used without awareness of their metaphoric origins and connotations, they nonetheless exert an influence on the direction of thought and emotional response. Talk about “nerves,” “tension,” or “stress,” for example, while often used to convey concern or distress in a colloquial way, prompts a search for internal or external sources of difficulty. “Nerves” points to internal vulnerability (Low, 1994), “stress” to a wide range of external circumstances (Young, 1980), and “tension” spans or bridges the two (Weaver & Karasz, 2022). Metaphors like tension have been widely adopted across cultures perhaps because (i) they are apt and readily understood through grounding bodily experience, (ii) they reference discourse endorsed by popular and authoritative sources in local and global media, and (iii) they are minimalistic or vague in their ontological assumptions or implications and thus have broad, flexible applicability.

Cultural illness explanations may also reflect metaphors rather than fully worked out models. The causal process or mechanisms that constitute the model may be characterized only by conventional names or labels that point to metaphors (or metonyms) without systematic elaboration or explication (Stern & Kirmayer, 2004). This is important because, without a detailed model, the explanation remains impressionistic, emotionally charged, and can easily convey unwarranted connotations. This happens with the diagnostic labels of psychiatry when they pass into everyday language. People sometimes use the adjective “schizophrenic,” for example, as a metaphor for being “split” in their thinking or pulled in two directions—a meaning that has little relationship to the phenomenology or mechanisms of the disorder. Terms like “tension” and “energy” may be used as experience-near metaphors that do not point to a causal mechanism though they presumably reflect underlying dynamics of attention, arousal, effortful engagement, cognitive dissonance, and other psychophysiological processes. Identifying these processes is crucial to understand the ways that metaphors contribute to embodied experience, symptom production, coping, and adaptation.

Metaphors can capture attention and amplify experience. For example, Gibbs quotes a woman with an eating disorder who says, “I need [my stomach] to be empty to feel alert.” This statement may reflect both a psychophysiological process involving gut–brain interactions (Mayer, 2011) and a metaphoric conceptual link between bodily states, emotion, and self-image.^8^ The contrasting feelings of emptiness and lightness or bloating and heaviness have a physical basis but provide natural metaphors for aspects of self-experience and evaluation that alter bodily habitus and self-regulation. Both psychophysiological and metaphoric elaboration then can contribute to cycles of symptom amplification with wider effects on behavior, identity, and social self-presentation. Another woman speaks of being pure and “sullying” herself with food. Again, the link between being sullied or dirty and consumption of food may be both metaphorical (e.g., eating is making a mess; i made a pig of myself) and reflective of a natural, physically based experiential link (hunger, eating, and satiety affect mood and cognition). Disturbed patterns of eating can lead to altered gastrointestinal function with feelings of gastric fullness, discomfort, or nausea (Murray et al., 2023). Bodily function and metaphoric construal form a loop so that the ways that patients and clinicians negotiate metaphors for experience can alter physiology and illness experience for better or for worse.

Even the most obdurate experiences have metaphoric mediation and elaboration. Gibbs discusses how metaphor intervenes in the sensory, affective, and cognitive dimensions of the experience of pain. The McGill Pain Questionnaire (MPQ; Melzack, 1975) provided a large vocabulary of English pain descriptors, most of which are clearly metaphors (Fernandez & Towery, 1996).^9^ The diverse language of pain serves to convey not only the sensory qualities of pain but its emotional quality and cognitive and social significance. Metaphors may not directly mediate the sensory intensity or “raw feel” of pain, but they certainly influence its evolution over time. Our endurance of pain depends in part on how we interpret its significance (Munday et al., 2022). Pain that is metaphorically evidence of injury or disease progression (“this pain is killing me”) can evoke fear and avoidance that lead to impairments in functioning (Löfvander & Furhoff, 1996).

Similarly, emotional pain, dysphoria, and distress have metaphoric mediation with both bodily and cultural roots. The term “depression” is used as an everyday idiom but also as an explanation for a wide range of dysphoric states, lack of hope, and behavioral withdrawal. As a metaphor, i am depressed implies being pressed down, as seen in the slumped posture, sense of heaviness or burden, and exhaustion of depression or demoralization. Both the psychophysiological state or habitus and its metaphoric self-construal may have global effects on how one engages the world, seeing others as more vigorous and energetic. This metaphor structure extends to the organization of perception and attention. For example, there is evidence that people with symptoms of depression tend to respond more quickly to objects in the lower rather than the higher portions of their visual fields (Meier & Robinson, 2006). Of course, depression has effects on levels of arousal, bodily regulation, and the impact of reinforcing stimuli that go beyond what might be expected based on the metaphor of being depressed. But the metaphoric elaboration of mood influences cognition, behavior, identity, and social interaction in ways that may create vicious cycles that amplify and maintain negative mood making it a more severe and persistent problem (Gómez-Carrillo & Kirmayer, 2023).

Just as they can amplify or exacerbate specific symptoms and sensations, metaphors can dampen or alter symptoms and distress. For example, reframing a symptom as a healthy or adaptive response or helpful communication (this pain is telling you to slow down; you are not depressed—you are burnt out and need a vacation/new job/new boss). Many therapeutic interventions can be seen as ways to introduce new metaphors for experience. Even the narrative arc of the therapeutic process itself may be structured by metaphor. For example, notions of catharsis, based on the metaphor of bodily expulsion, imply that there is something toxic inside the person (negative emotion, traumatic memory, suppressed conflict) that must be released. The therapeutic ritual or conversation aims to achieve this expulsion. In its etymology, the metaphor of catharsis does not capture the complex dynamics of emotion regulation, which involve narrative (re)framing, imagery, and enactment. But the concept of catharsis was elaborated in psychoanalysis and other therapeutic approaches by drawing from theatre and other ritual practices to include the notion of an optimal aesthetic distance that allows the expression of emotion in ways that produce a sense of release (Scheff, 1979). Aesthetic distance itself can be regulated by metaphor. Metaphors can create distance—whether because of the way they position us vis-à-vis the situation they describe (this is an old story; it's just a bad movie) or because we become aware of our construal as metaphorical and hence become free to re-frame or aestheticize experience. To understand how this transformative function of metaphor works, the metaphoric mediation of illness experience and healing practices must be unpacked through detailed attention to local cultural systems of meaning and practice, which include sensory worlds, ontologies, social institutions, and overarching narratives. Many of the papers published in this issue of *Transcultural Psychiatry* provide instructive examples of this unpacking.

## Illness experience, ontology, and explanation

Recent work in anthropology and sociology has advocated for an ontological turn in which cultural systems are recognized as constituting fundamentally different realities (both experiential worlds and material orders) in which humans and other beings are deeply intertwined in ways that are not adequately captured by the usual divides of culture and biology (Descola, 2013; Kohn, 2015; Latour, 2013; Viveiros de Castro, 2004). Taking cultural ontologies seriously requires considering the ways in which ontologies arise from specific modes of material and discursive practice (Hacking, 2002; Pickering, 2017).

In their contribution to this issue, Sass and Alvarez (2023) engage with the debate about ontology through their reflections on ethnographic research with practitioners of an Indigenous healing system. They describe some key concepts of mental or emotional disorders recognized by healers (*curandera*) among the Mexican Indigenous Purépecha people. They explore both the metaphoric meaning and underlying ontology of *locura* (madness), *nervios* (nerves), and *susto* (fright illness). The Purépecha distinguish “good” illnesses that have physical or natural causes and “bad” illnesses that reflect supernatural, magical, or malign effects of witchcraft and spirits. Bad illnesses often have a mental or psychological quality associated with emotionality.

Sass and Alvarez invoke Michel Foucault's (2005) analysis of epistemes in *The Order of Things* and relate these to different ontologies. Foucault argued that the medieval and Renaissance episteme was grounded in the concept and experience of “similitude” of which four varieties were recognized: (i) *Convenientia* is based on physical proximity (contiguity or metonymy); (ii) *Aemulatio* involves perceptual resemblance (physiognomy or appearance, metaphor); (iii) *Analogy* reflects structural parallels (synecdoche); and (iv) *Sympathy*, which is based on similarity of effect or impact on the person. This provides the epistemic grounding of magic.^10^ This episteme is experienced not just as a valid, trusted, way of knowing but also as an embodied ontology—entities, agents, presences, and facts about the world that one can feel, and experience in self-vindicating cycles of action and perception. Sass and Alvarez (2023) suggest that this approach can help ground recent efforts to better appreciate and characterize differences in ontology across cultures to appreciate “their depth, encompassing scope or constituting power” (p. 784). They compare the ontological dimension of human experience to a “mood-like state.” In computational models of cognition, this might appear as a prior (or expectation) not just in a system of causal modelling/attributions or perceptual templates, but as a broader anticipatory stance and affective ambience.

This is an important shift from ontology as a predicate-like assertion of what the world consists of or contains (gods, spirits, animals, atoms, quarks) to an affective stance that precedes and pervades our engagements with the world. We can take this further. An embodied-enactive perspective would suggest that we experience the world as harboring supernatural entities not only because we observe things moving or acting “on their own” and have feelings of “presence,” but also because we engage in practices like prayer, curses, and magical spells; that is, there is an implicit ontology that resides in the loop between perception and action. Ontologies then may be experienced and enacted through practices. The active inference or predictive processing model suggests that the most basic features of ontologies are the expectations that guide attention, generate sensations of perceptual presence, and govern attributions of agency and constraint. These are embodied experiences that can exist prior to the elaboration of any particular metaphysics. Making these ontologies explicit in metaphysics, mythology, or other cultural models and metaphors adds an additional linguistic loop to the more basic embodied engagement.

Tidwell et al. (2023) describe how a cultural ontology shapes Tibetan medical perspectives on affliction. They focus on cases of spirit possession that are grounded in a cultural system in which “humans share the natural world with a range of spirits and deities that inhabit rivers, land, and mountains, and govern natural systems such as weather patterns, karmic retribution, fertility, growth, wealth, and so forth” (p. 806). The first case they discuss is a woman who sees ghosts and is agitated and disorganized. She is given treatment—presumably exorcism—as indicated by suddenly having a new name inscribed on her chart. The name change itself is an intervention that reflects a larger ontology:

Ritualized name changes and related purifications help remove obstacles for an individual through establishing a new set of karmic connections. All who knew her would start calling her by this new name, helping to solidify this new set of relations. (p. 805)

Tidwell and colleagues explore this process of affliction and healing in terms of the ways that cultural affordances shape bodily experience and symptom interpretation (Ramstead et al., 2016).

Of course, metaphors are also the vehicle through which we develop theory and Tidwell et al. (2023) use the metaphor of kindling to capture the ways in which repeated experience can increase the propensity for some particular kinds of symptoms. The idea of kindling has been used in the study of epilepsy, where repeated seizures lead to neurophysiological changes that increase the propensity for further seizures. The metaphor of kindling a fire seems apt when we are describing a paroxysm of electrical activity, where the effects of a seizure spread much like a fire and transform the substrate (regions of the brain) that are affected. Luhrmann (2020) has suggested that a kindling process can help explain how religious practice can alter sensory experience, giving rise to experiences of speaking in tongues or hearing the voice of God and could also explain aspects of phenomena like auditory hallucinations (Luhrmann et al., 2015). Tidwell et al. (2023) suggest that a similar process of kindling constitutes a kind of bio-looping in which cultural categories and classification reshape bodily experience to give rise to the dissociative phenomena of *dön* possession. They speak of “kindling of cultural affordances, where the threshold for interpreting physiological cues” as a particular kind of symptom or malady “is lowered through cultural exposure” (p. 810). The metaphor kindling in this instance relates to the likelihood of an interpretation rather than of a bodily process. We might ask when is it useful to substitute *kindling* for *learning*, *interpretation*, or for a computational metaphor or model that involves changing expectations in a predictive processing system? To characterize the kinds of learning involved in the more skilled and scripted practice seen in dissociation, the metaphor of kindling, which implies developing a subpersonal embodied propensity akin to a lowered threshold for seizures, may be less revealing than other metaphors, e.g., (i) developing new expectancies or priors in a Bayesian brain; (ii) developing a way of perceiving and responding to the cultural affordances in an environment; (iii) learning to occupy a social niche or position with a particular habitus; or (iv) laying down a path through a socially configured landscape that canalizes subsequent behavior (Seligman & Kirmayer, 2008).

The choice of metaphors is important not only because it suggests where we might look for the underlying mechanisms of disorder but also because Tidwell et al. (2023) argue that the bio-looping version of kindling can help explain the fact that the phenomena seen in the Tibetan settings they study can move across cultural, religious, and ethnic boundaries to affect people from different backgrounds. They suggest that we think in terms of “transcultural” affordances that can work across cultural contexts. What might be the grounding of such transcultural affordances: a physiological process of kindling, existential/experiential universals, cultural diffusion, or reinvention? The answer to this question has implications for clinical work in cultural psychiatry with people from diverse cultural backgrounds, languages, and communities who co-exist in multicultural communities and urban centers.

Here again, metaphor theory offers some insights. Kövecses (2005, 2015) discusses the cultural origins of metaphors and identifies multiple sources including bodily experience, existential predicaments, and cultural-linguistic histories. Experiential universals can yield “natural” metaphors without a particular ontology. For example, Tidwell et al. (2023) note:

The idea of wind as a medium for demonic afflictions or exogenous pathogenic factors is transferable across ethnic, cultural, and medical boundaries … Rebkong's local landscape of shared ontologies across ethnic and religious boundaries inculcates shared expectations, or regimes of attention, to animate illness experience and inform therapeutic behavior. (p. 810)

Everyday experiences with wind may provide a natural way to think about the impact of the environment on the person. This could be described in terms of bodily grounded *ecological affordances*. However, linking the wind to demons clearly involves a specific cultural ontology. We need a notion of ontology that does not necessarily reflect an explicit metaphysical model or theory but that is grounded in practices. These practices are usually part of local cultural systems, but they can also be borrowed, blended, or hybridized from diverse traditions, and repurposed in multicultural settings. As Tidwell et al. suggest, bio-loops of action and perception that involve physiology, imagination, and cultural affordances present in the environment provide a way of thinking about ontology as embodied and enacted practice.

## Transforming symptoms and suffering

The idea that healing rituals and practices transform illness experience through metaphoric mapping recurs in much ethnographic writing on healing. Most famously, Levi-Strauss (2003) gave an account of how healing might occur through mapping the painful body onto a mythic realm. Although the ethnographic details of the example he used render his original account problematic (Kirmayer, 1993), the structural approach he provided remains compelling. What is needed to make the account work is closer attention to the communicative and mutative effects of language on physiology, cognition, and self-regulation as well as on the social-rhetorical processes of persuasion, self-presentation, and positioning.

Anthropological studies of healing rituals have tended to approach them as coherent structures in which every detail converges on a shared meaning that reinforces collective values and institutions, reinscribing the affliction within a local cosmology and reinserting the person into a local world. Attention to the aesthetics and pragmatics of ritual illustrates cultural creativity but is seen as in service to maintaining the social order. Psychological studies of healing look more closely at the microdynamics of cognition and communication which offer alternative ways of (re)structuring action and experience. These same processes can be seen in diverse forms of psychotherapy, which may make explicit use of metaphors.

Metaphors can be used in diverse ways in psychotherapy (Barker, 2013; Cox & Theilgaard, 1997; Mathieson et al., 2020; McMullen & Tay, 2023; Tay, 2022; Törneke, 2020). Therapists may work within a client’s metaphor, seeking to extend it in ways that open up new interpretations and adaptive possibilities. They begin by adopting the metaphor, then modify it to modulate its intensity, rein in some of its problematic implications, and set the stage for an alternate metaphor that may be more accurate or more salutary and salutogenic.

Seligman (2023) explores the relationship between metaphors and emotion in the context of adolescent distress and psychotherapeutic treatment. She draws from an ethnographic study of Mexican American adolescents receiving outpatient treatment for a variety of emotional and behavioral problems, to consider both the metaphors that adolescents use to make sense of their experience and the ways they respond to the “prescribed” metaphors deployed in mainstream, manualized child and adolescent Cognitive Behavioral Therapies used in these clinical settings. Seligman uses the term “prescribed metaphors” to point to the way these metaphors are medically authorized and presented as explicit interventions to reshape patients’ thinking and invoke social and cultural scripts for appropriate behavior. Choudhury et al. (2015) described a similar process in the ways that adolescents are encouraged to make sense of the prescription of psychiatric medications as vehicles for emotion regulation.

Seligman (2023) describes adolescents’ responses to the use of an “emotion thermometer” to graphically record the intensity of their feelings. While some found the metaphor of intensity easy to map onto the visual analogue scale of the thermometer, others protested that the task was difficult and constraining because their feelings fluctuated, were situation dependent, and involved multiple qualities or dimensions. The pan-cultural metaphor of anger is heat was transformed in youths’ narratives into a more dynamic account of struggling to contain, manage, and express strong feelings. In some instances, youth rejected the injunction to moderate or reduce emotional intensity, recognizing that strong emotions serve a moral function: anger is a sign of the recognition of injustice, a way to communicate that recognition, and becomes an armature around which an identity as an aggrieved, unfairly treated, and righteous person can be constructed. The instruction to monitor feelings, which is aimed at creating some meta-cognition, distance, and emotional self-regulation may then be perceived as undermining a familiar, socially justified, and legitimate sense of self and moral conviction.

In Seligman’s (2023) ethnographic account, adolescents found some metaphors apt and readily took them up, while they resisted or refigured other metaphors through acts of poiesis: “Some metaphors may stick, while others may not, depending on what they do for the individual—both intra and interpersonally” (p. 821). What makes a metaphor apt may be its fit with embodied experience, existing models, or over-arching narratives that have institutional authority—and hence, its effectiveness to make sense of or manage feelings both intrapsychically and interpersonally. This interplay between daily experience and culturally salient narratives is evident in accounts of many kinds of symptom experience. For example, acts like self-cutting may exploit aspects of bodily physiology through which injury activates endorphin systems that reduce pain and anxiety (Kirmayer & Carroll, 1987; Kirmayer, 2008). In addition to this physiological response, self-injury is ascribed symbolic meaning—both in the moment and on later experiences with scars—that loops back into its use as a coping strategy and act of communication, identity construction, and social positioning. Through such bio-looping, metaphors do not simply describe experience, they reshape it. Emotions, then, are not fixed entities that dictate specific metaphors but embodied and socially situated experiences that are differently constructed personal and cultural contexts (Barrett, 2017). Part of this construction is mediated by metaphors. Through metaphor, emotions are embodied, embedded, enacted, and encultured in the social world.

## Colonialism and the politics of language

Saussurean linguistics argued that the links between words and things, objects and signs is arbitrary (Hawkes, 2003). But for the poet—no less than the patient struggling to express their predicament—this relationship seems much less arbitrary. Though there is room for playful invention and improvisation, the connection between words and things feels inborn, basic, or primordial. Poets are rooted in the specificity of their language. Languages, in turn, are rooted into specific worlds of experience. The metaphors we use to articulate everyday experience have a mundane, commonsense quality that tends to obscure the fact that they are always inscribed in particular histories. In most instances, these histories are not just local but global. They may the traces of migration, colonization, conflict, intermixing, and enduring structures of oppression or of privilege. Becoming aware of these histories can serve important therapeutic functions (Kirmayer, 2022; Walsh, 2022).

Colonization served to negate local languages, knowledges, and sacred orders. It installed the language of the colonizer as the source of power, truth, beauty, and moral rectitude. Colonial subjects pushed back against this domination and erasure, bending the colonizer's language to their own ends. Generations after decolonization, this effect of linguistic domination has persisted in the devaluing not only of Indigenous languages but also of the creoles that naturally emerged through the collision of languages and traditions. The poet, critic, and philosopher, Édouard Glissant (1997) spoke powerfully for the importance of mixing or métissage and the vitality of creoles and other hybrid cultural and linguistic forms as the basis of creativity not only in the Caribbean but in a globalized world (Glissant, 2020). One expression of this creativity is the development of culturally grounded modes of therapy.

In their contribution to this issue, Walcott et al. (2023) describe the application of Psychohistoriographic Cultural Therapy (PCT), a method of group therapeutic intervention developed by Frederick Hickling in Jamaica (Hickling, 2012, 2020, 2021). PCT starts from the recognition that individual and group interactions are embedded in a cultural matrix that reflects local histories and gives rise to particular identities. In Jamaica, this matrix includes the history of colonialism, enslavement, and postcolonial independence. Individuals and groups are shaped by this history, which provides a shared horizon, social structure, and implicit background knowledge. Becoming aware of where one is situated in relation to this structure and background through a process of dialogue and dialectical analysis can provide a way to recognize, transform, and transcend the legacy of oppression.

The dialectical process of PCT identifies polarities, oppositions or antipodes that give rise to the different positions of group participants and the larger cultural-historical frames in which they are embedded. This process reveals multiple dimensions of identity, interrelationships between seemingly unrelated identities, their mutual constitution (e.g., the dialectic of master/slave), and dependence on a shared history and social space. Group participants locate themselves in this process and portray aspects of their experience with poetry, song, and drama. These are assembled into a performance that provides a symbolic container or aesthetic ordering of the tensions within the group and the larger society. While these tensions may be difficult or intolerable in everyday life, art-making processes can create a symbolic order and container where tensions and contradictions can, at least temporarily co-exist. Of course, facilitating this process requires skills in mediation because problems can arise when the symbolic container is not sufficient, or the essential process of mutual recognition is inadequate (Hickling et al., 2010).

The creation of a group performance—which may include visual art, music, dance, and poetry—serves to articulate, acknowledge, and explore these facets of identity. Presenting participants’ diverse identities together, in interaction with each other, allows a degree of containment and wholeness, even when the tensions cannot be completely resolved. In a sense, the group process of poiesis provides a map and container for the tensions that constitute a society or diverse community. The use of the arts reflects their unique power but also, in this case, their valorization within Jamaican culture as an icon or emblem of collective identity.

Walcott et al. (2023) show how this culturally informed method of exploring group tensions can be applied to working through conflicts in everyday life. Although they use psychiatric terminology to characterize personality traits and maladaptive behaviors that undermine group collaboration, the recognition of historically constituted forms of subjectivity and identity allows Walcott et al. to analyze conflicts in ways that go beyond conventional group therapy and provides metaphors that can be deployed in therapeutic interventions to move individuals and the group toward more adaptive modes of working together. They use the Rastafarian metaphor of overstanding to describe the insights that arise from exploring the ways that everyday structures of inequity are played out in individuals’ adaptation and group process. Metaphor and *poiesis* then become crucial resources for decolonizing practice in therapy and other social contexts.

## Metaphor as pedagogy and practice

Explicit attention to metaphor can provide a critical perspective on medicine with rich potential for clinical pedagogy and practice. Medical language is replete with metaphors that convey surplus meaning or connotations that shape clinical thinking, communication, and practice (Bleakley, 2017; Fuks, 2021; Kirmayer, 1988, 1992). Given the centrality of metaphor in everyday cognition, experience, and communication, it is not possible or desirable to expunge medicine of metaphor. The task, instead, is to develop a critical awareness and sensitivity to hidden or implicit connotations of dominant metaphors and, when they appear harmful, to use our poetic and imaginative faculties to develop and deploy alternate metaphors.

Arlene Katz (2023) discusses an approach to medical education that aims to help physicians better grasp the nature of patients’ lifeworlds through a process she calls *social poetics* (Katz & Shotter, 1996). This is a participatory process that involves creating a space in which patients’ lived experience and life stories can be told. As she illustrates, clinical discourse focuses on translating elements of personal history, symptoms and signs into diagnoses, causal explanations, and corresponding interventions (Mishler, 1984). Social poetics encourages and attends to conversations in other registers, narrative forms or genres more closely aligned with patients’ everyday understanding, self-presentation, and negotiation of their lifeworlds. In addition to the social practice of creating space for and valuing patients’ stories, this requires learning to listen to language as embodied in a particular lifeworld with its own sensory and sensual qualities and becoming alert to the strategic or creative moves that are made through poiesis—introducing new metaphors that convey concerns and evoke a response from others.

There is an intimate connection between attentiveness and caring. The effort to care for another in ways that respond to their lived experience demands close attention. Attentiveness itself is a form of care for the other and nurtures one's own depth and moral presence (Kleinman, 2020). Attention fosters awareness and concern centered on the minute particulars of another's experience. This begins in dyadic interactions with empathic listening. Understanding the other requires learning about their lifeworld. Given the ways we are differently embodied and embedded in local worlds, taking clinical interaction beyond the moment-to-moment effort to track an illness narrative to a deeper appreciation of lived experience requires an ability to imaginatively inhabit the other's social world, which includes material circumstances and relationships with specific people. Katz (2023) notes that an important key to this exploration is the experience of surprise, which allows us to recognize and address it with respect and willingness to learn. This extends to recognizing the larger social, cultural, and historical context that imbues language and the environment with meaning and creates structures that matter. Hence, the clinical dyad is embedded in a triadic relationship, in which others in the social world provide a matrix of meaning and possibility.

Similar concerns with attentiveness, caring, and understanding motivate the article by Hanna De Jaegher (2023), which considers the ethical and epistemic challenge of understanding others who may have substantially different experience and modes of interpersonal interaction that pose specific challenges to communication and mutual understanding, like people with autism.^11^ De Jaegher (2023) brings together insights from the neurodiversity movement, enactive cognitive science, and Indigenous epistemologies to underscore the ethical and pragmatic value of engaging with others through a process of “letting be”—a concept she draws from the work of MacLaren (2002). De Jaegher (2023) enlarges this with the recognition from enactivism that “most of sense-making is both participatory and situated” (p. 856). Indigenous and other postcolonial perspectives insist on the fundamentally political nature of the process of knowing.

As a mode of knowing, letting be is something other than co-existence. To implement this, De Jaegher (2023) recommends we start from “where and how people are… and let them be where and how they are” (p. 859). However, this formula presents conceptual and practical challenges. In so far as we need others to scaffold our experience, simply “letting be” in the sense of remaining neutral or non-interfering may not allow the other to be as they usually would or want to be. To know other people, we must be present and responsive to them, interacting in ways that do not silence or dominate but provide them with the interpersonal affordances needed to elaborate their experience. In effect, we must remain open to the other and allow ourselves to be affected and transformed by them in ways that, coupled with self-awareness, enable us to know and understand aspects of their lifeworld (Kirmayer, 2015). Knowing as *letting be* then means letting be *with* another, co-constructing and inhabiting a shared world or meeting place, and *letting go*—that is, allowing oneself to become someone different in some (significant) way. Knowing is embodied, embedded, and extended over time and space in ways that are experiential, relational, and instrumental: as a way of being in the world, a way of being with others, and a way of acting in the word with specific goals or purposes. As knowers, we are never disinterested—so an ethic that elevates or prioritizes the other is essential to avoid occluding them with our own desires and agenda. All of this points to the importance of metaphor in articulating modes of being and an ethical stance that acknowledges otherness or alterity (Kirmayer, 2007). This includes recognizing the provisional nature of our metaphors and the humility to acknowledge what we do not know (Kirmayer, 2013).

The ethics of alterity and the limits of knowing are also central to the article by Hiba Zafran (2023). She describes her experiences working to incorporate Indigenous perspectives in health science training in response to the calls to action of the Truth and Reconciliation Commission of Canada (2015), which documented the devastating effects of Canada's policies of forced assimilation through state-mandated Indian Residential Schools. Assigned the task of moving her academic program toward greater inclusion of Indigenous applicants, histories, and healing practices, Zafran and her colleagues focused on developing cultural safety from the bottom-up, as a kind of “micro-reconciliation” (Tait et al., 2019), at the level of individual participants in educational activities that will hopefully prompt changes in institutional policy, training, and clinical practice.

Zafran (2023) reflects on her own complex identity and positionality as she engages with Indigenous knowledges and silenced voices through advocacy, allyship and friendship with colleagues, but also through a process she calls *echopoetics*, assembling fragments of others’ language into “found poetry” that echoes (and transforms) her experience and concerns. In echoing the words of others, she recognizes their voices and blends them with her own.

When we listen to the world, we sometimes hear only echoes: the world seems to mirror our desire (or taunts us with distorted echoes of our own voice). What transforms such *echopoetics* into *ecopoetics* is our recognition of other voices in the world that present the unexpected and unimagined. This engagement can re-animate and re-populate our imagination with new metaphors and perspectives in surprising and unanticipated ways. The poet-philosopher Jan Zwicky (2014) has offered a lyric philosophy that highlights this process of poetic refiguration as the basic way we encounter and respond to the world. Lyric philosophy taken into the world (or, better, inhabiting the world) becomes *lyric ecology* (Dickinson & Goulet 2010), an approach that holds the promise of bridging the nature/culture divide by insisting on the ecological embedding of poiesis.

## Conclusion: Knowledge, aesthetics, and community

History and literature tell us a great deal about the human mind—not because they are inside it but rather because it is inside *them*. (Bringhurst, 2008, p. 237)

In this paper, I have sketched an approach to the poetics of illness experience and healing centered on the role of conceptual metaphor. We think with and through metaphors that we learn from our families and cultures. Metaphors structure our everyday experience as well as the technical models we use to understand illness and healing. Metaphors can organize inchoate experience, promote creative thinking and problem-solving, and enliven or challenge our conventional or commonsense views. Common sense itself is a cultural construct that makes some metaphors seems natural or inevitable. Critical analysis of the metaphors in which psychiatric theory is framed and applied can reveal dilemmas created by our language and encourage the development of new metaphors that more accurately describe the texture of experience and the processes of illness and healing.

Language is a vehicle for connection, presence, and co-presence, but also for misunderstanding, distance, and disconnection. Poiesis draws attention to the ways in which experiences that escape narrative containment can be expressed and explored. Learning to attend to the metaphors through which patients articulate experience allows us to see the interplay between personal and cultural meaning. Attention to poetics in cultural psychiatry can enhance empathy, clarify the sources of suffering and impairment, and provide resources for helping and healing interventions.

## Notes

1. In addition to the authors of papers in this issue, other presenters at the ASI included: Debbie Ann Chambers, Jaswant Guzder, Bradley Lewis, Omri Moses, Melissa Park, Cécile Rousseau, Brad Shore, Somogy Varga, and Samuel Veissière. Tawni Tidwell did not attend the workshop but her paper is included because of its relevance to the theme.

2. Following a common convention, metaphors under discussion are formatted in small caps.

3. The idea of semantic pointers allows for multiple types of meaning-laden content in cognition (Blouw et al., 2016). For a discussion of metaphoric meaning in terms of semantic pointers, see Thagard (2022).

4. In the Bayesian computational modelling of active inference, priors are the expected values of a predicted outcome before any new information (Parr et al., 2022); priors can be revised based on information, or higher order changes in expectation. Cultural contexts and practices can install higher order priors through explicit communication and implicit learning (Veissière et al., 2020).

5. Resources for developing a neural or computational theory of conceptual metaphor can be found in earlier work on computational modelling of linguistic metaphor (Kintsch, 2000), neurobiological research on language and metaphor (Beaty et al., 2017; Desai, 2022; Feldman 2008; Lakoff, 2008, 2014); semantic pointer theory (Eliasmith, 2013; Thagard, 2022), and more recent predictive processing or active inference approaches (Bouizegarene et al., 2020). While the active inference approach provides a generic version of cognitive structure, the neural theory of metaphor invokes specific brain structures and processes. Hence, the associations in metaphor are not arbitrary but reflect: (i) sensory similarities or cross-modal links through synesthesia; (ii) assessments of affective meaning (extending Osgood's E-P-A (Osgood, May, & Miron, 1975) to other culturally grounded, neurally constructed aspects of affect and emotion (Barrett, 2017); and (iii) topographic connections associated with the structure of neural representations of action and perception. To this we can add the developmental learning and top-down influences of cultural scripts and scenarios that forge additional links. The key point is that metaphoric associations are not arbitrary but deeply rooted in the structure of the nervous system (and the body) as well as the socially constructed worlds we inhabit.

6. Because they are rooted in personal and cultural histories, the use of familiar or shared metaphors can create intimacy (Cohen, 1978). However, when people are embedded in multiple cultural strands or contexts, the use of metaphors can be part of linguistic code-switching that marks differences in identity and position, creates boundaries, and demands a shift in perspective (Auer, 2013).

7. Slingerland (2004, 2008) has argued that conceptual metaphor theory and embodied cognitive science provide resources for understanding cultural and historical variations in religious ontologies and practice.

8. For a discussion of the regulation of subjective experiences of tension, energy, arousal, and restlessness, see Thayer (2003).

9. Semino (2017) found that some of the terms in the MPQ are not common in everyday usage.

10. The operation of “sympathy” or similarity and contagion in disgust and other affective responses is well documented (Rozin & Nemeroff, 1990).

11. Or “autistics”—the choice of language reflects both different identities and particular socio-political positions (Anderson-Chavarria, 2022; Cascio, 2020).
